# Spectral Diversity and Regulation of Coral Fluorescence in a Mesophotic Reef Habitat in the Red Sea

**DOI:** 10.1371/journal.pone.0128697

**Published:** 2015-06-24

**Authors:** Gal Eyal, Jörg Wiedenmann, Mila Grinblat, Cecilia D’Angelo, Esti Kramarsky-Winter, Tali Treibitz, Or Ben-Zvi, Yonathan Shaked, Tyler B. Smith, Saki Harii, Vianney Denis, Tim Noyes, Raz Tamir, Yossi Loya

**Affiliations:** 1 Department of Zoology, Tel-Aviv University, Tel-Aviv, Israel; 2 The Interuniversity Institute for Marine Sciences of Eilat, Eilat, Israel; 3 Coral Reef Laboratory, University of Southampton, NOCS, Southampton, United Kingdom; 4 Institute for Life Sciences (IFLS), University of Southampton, Southampton, United Kingdom; 5 Department of Biotechnology Engineering, Ben-Gurion University, Beer Sheva, Israel; 6 Charney School of Marine Sciences, University of Haifa, Haifa, Israel; 7 Center for Marine and Environmental Studies, University of the Virgin Islands, St. Thomas, United States Virgin Islands, United States of America; 8 Tropical Biosphere Research Center, University of the Ryukyus, Motobu, Okinawa, Japan; 9 Biodiversity Research Center, Academia Sinica, Taipei, Taiwan; 10 Bermuda Institute of Ocean Sciences (BIOS), St. George's, Bermuda; 11 Faculty of Life Sciences, Bar-Ilan University, Ramat-Gan, Israel; King Abdullah University of Science and Technology, SAUDI ARABIA

## Abstract

The phenomenon of coral fluorescence in mesophotic reefs, although well described for shallow waters, remains largely unstudied. We found that representatives of many scleractinian species are brightly fluorescent at depths of 50–60 m at the Interuniversity Institute for Marine Sciences (IUI) reef in Eilat, Israel. Some of these fluorescent species have distribution maxima at mesophotic depths (40–100 m). Several individuals from these depths displayed yellow or orange-red fluorescence, the latter being essentially absent in corals from the shallowest parts of this reef. We demonstrate experimentally that in some cases the production of fluorescent pigments is independent of the exposure to light; while in others, the fluorescence signature is altered or lost when the animals are kept in darkness. Furthermore, we show that green-to-red photoconversion of fluorescent pigments mediated by short-wavelength light can occur also at depths where ultraviolet wavelengths are absent from the underwater light field. Intraspecific colour polymorphisms regarding the colour of the tissue fluorescence, common among shallow water corals, were also observed for mesophotic species. Our results suggest that fluorescent pigments in mesophotic reefs fulfil a distinct biological function and offer promising application potential for coral-reef monitoring and biomedical imaging.

## Introduction

**Fig 1:**
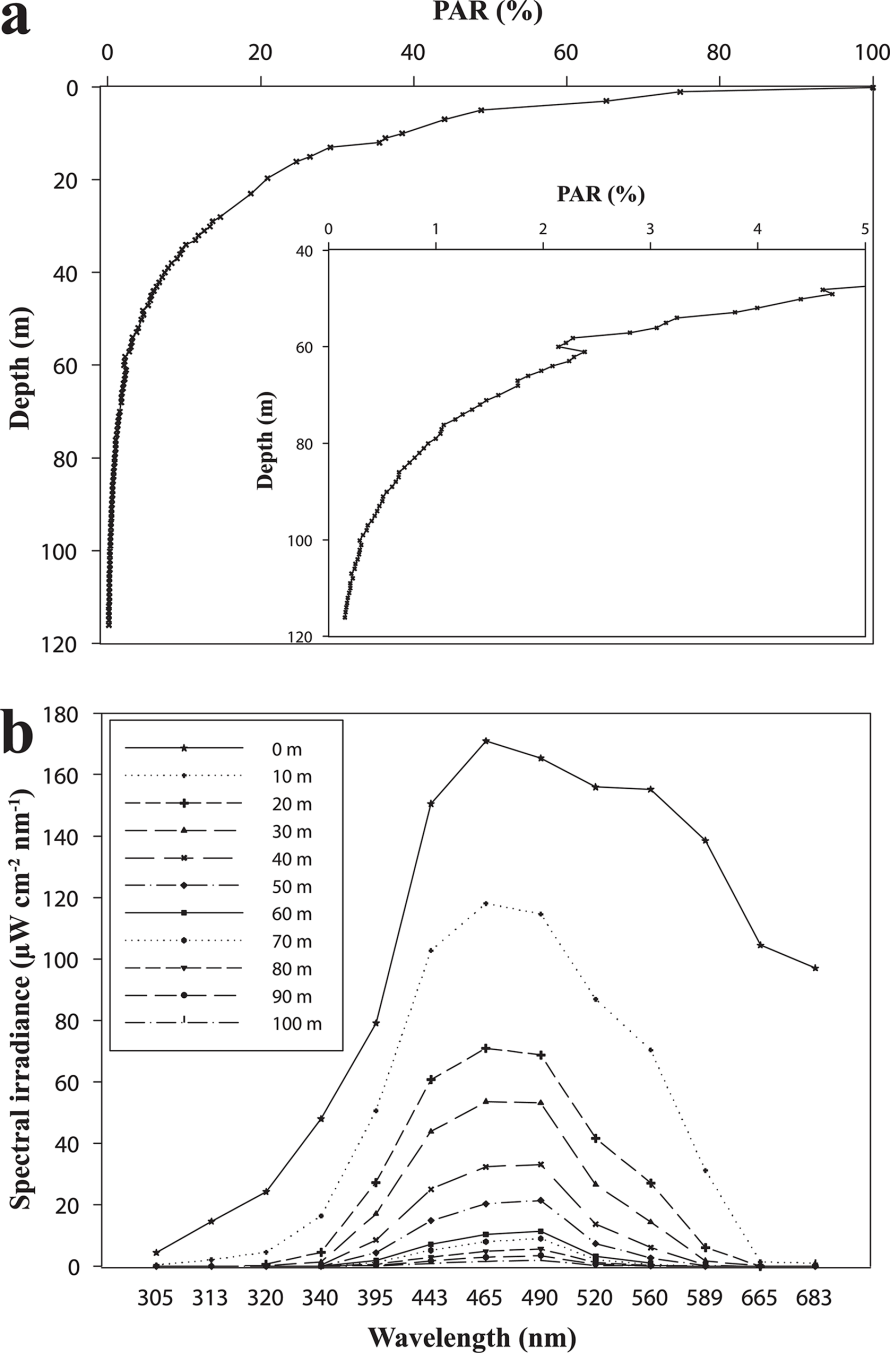
Changes in downwelling photosynthetic active radiation (PAR) and spectral composition of the underwater light in different depths in the Gulf of Aqaba off the Interuniversity Institute in Eilat on the 29^th^ October 2015. (a) Percentage of PAR at various depths expressed as % of surface PAR (1400 μmol*m^-2^*s^-1^). The inset shows the values for mesophotic depths. (b) Depth-dependent changes in spectral irradiance.

The obligatory symbiotic association with photosynthetic algae of the genus *Symbiodinium* (zooxanthellae) renders the major builders of shallow, warm-water reef scleractinian corals, dependent on sufficient amounts of photosynthetically-active radiation (PAR) [1–4]. Both the intensity and spectral composition of the irradiating sunlight change dramatically with increasing water depth [5–7]. Ultraviolet and red wavelengths are gradually removed from the spectrum resulting in a blue-green underwater light field at greater depths (Fig 1).

In the shallowest parts of a reef, corals and their zooxanthellae often experience stress due to excessive light exposure. With increasing water depth, light becomes limiting rather quickly for the coral-zooxanthellae symbiosis [5,8,9]. Zooxanthellate corals are often most abundant and diverse at depths down to 30 m [10,11]. However, numerous species also occur at mesophotic depths (30–150 m), among them *Leptoseris fragilis*, with a distribution that extends to a maximal depth of ~145 m [11]. Light can thus be considered one of the most important environmental factors structuring coral-reef communities, confronting both the coral host and the algal symbiont with a range of physiological challenges. For instance, mesophotic reef corals live at depths at which high light stress is not relevant, but in which their algal symbionts need to succeed under a limited supply of PAR. Fluorescence has been suggested to enhance the performance of zooxanthellae in low-light environments at depths down to 40 m [12]. Interestingly, “light-harvesting” by fluorescent chromatophores has been found to extend the distribution of the zooxantellate coral *L*. *fragilis* to depths beyond 100 m in the Red Sea [6]. In this coral, as well as in other deep-water representatives of the family Agariciidae from the Red Sea, autofluorescent chromatophores were found in the oral gastrodermis emitting brilliant blue/turquoise fluorescence (between 430 and 500 nm wavelength) under excitation with light at 365–410 nm [13,14]. The chromatophores are organised in dense layers underneath the zooxanthellae and are thought to transform the irradiating light from shorter into longer wavelengths, thereby enhancing algal photosynthesis. Some of the pigments could be extracted in chloroform, but the biochemical nature of the chromophores is as yet unknown [6]. The fluorescence of colour morphs of *Leptoseris* spp. from mesophotic depths in Hawaii could be assigned to chromophores with spectral properties indicative of the presence of cyan and green fluorescent representatives from green fluorescent protein (GFP) family [15].

In shallow-water corals, GFP-like fluorescent proteins are among the most prevalent pigments [16]. Together with the biochemically closely-related non-fluorescent chromoproteins (CPs), these fluorescent proteins (FPs) are responsible for most of the green, orange-red and purple-blue colours of reef corals [16]. The colour diversity arises from variations in the chromophore-forming amino acid triplet and the interactions of the chromophore with the surrounding protein scaffold [17,18]. In many shallow-water corals, the production of the pigments is regulated at the transcriptional level by light, in particular from the blue spectral range [19]. A light-driven upregulation of FPs and CPs is also involved in growth-related processes such as repair of mechanical damage or defence against epibionts [20]. Other environmental factors including temperature stress can also affect the expression of FPs and CPs [21–23]. The genes coding for these proteins occur in multiple copies in the coral genomes and the striking colour polymorphisms of reef corals are associated with large intraspecific differences in expression levels resulting from variation in the number of actively expressed genes [24].

Both FPs and CPs have been suggested to function as sunscreens for the corals and their symbionts [25–28], and recent experimental studies have provided evidence of their ability to reduce photo damage in zooxanthellae under acute light stress [24,29]. FPs have also been found in corals such as *Montastraea cavernosa*, *Lobophyllia hemprichii* and *Galaxea fascicularis* [30–33] which typically grow in lower-light habitats. In these species the expression of the pigments is less dependent on the intensity of irradiating light compared to that of acroporids from shallow-water habitats [24,31,32]. The differences in gene regulation support the notion that GFP-like proteins fulfil multiple functions in different light environments [19,32].

Up to now, the role of fluorescence in corals in low-light habitats at depths >40m has remained largely unstudied due to the difficulties in accessing these habitats using standard SCUBA diving. Recent advantages in technical diving approaches [34,35] and fluorescence detection techniques [36–38] facilitate the exploration of coral fluorescence in mesophotic corals. Here, we provide first insights in the prevalence, spectral properties and regulation of coral fluorescence in a mesophotic coral community in the Red Sea in Eilat, Israel.

## Materials and Methods

### Coral collection

Coral samples were collected under the permits No. 40613, No. 40621, No. 40230, No. 38726 and No. 38249 issued by the Israel Nature and Parks Authority. Sixteen mesophotic stony coral species, comprising *Acropora squarrosa*, *Alveopora allingi*, *Alveopora ocellata*, *Blastomussa merleti*, *Echinophyllia aspera*, *Euphyllia paradivisa*, *Favites cf*.*bestae*, *Goniopora minor*, *Goniopora somaliensis*, *Leptoseris glabra*, *Leptoseris* sp., *Oxypora egyptensis*, *Pleuractis granulosa*, *Stylocoeniella guentheri*, *Stylophora pistillata*, *Turbinaria reniformis*, and two corallimorpharians: *Discosoma unguja* and *Rhodactis rhodostoma*, were collected from 50–60 m depth off Dekel Beach (29°32'20.02"N 34°56'44.80"E) and the IUI (29°30'6.19"N 34°55'3.64"E) for examination of their fluorescence spectra. In addition, four dominant shallow coral species: *Acropora* sp., *Hydnophora exesa*, *Pocillopora damicornis* and *Stylophora pistillata*, were collected from 1–25 m depth off the IUI. One to three fragments (~10 cm) collected from different colonies were photographed *in situ* with a standard underwater camera and with a fluorescence imaging system which was composed of a standard Cannon 5DII SLR camera with a Tiffen #12 yellow filter and Xenon strobes fitted with blue filters for illumination [37]. Fluorescent images of *G*. *somaliensis* were taken *in situ* using the excitation produced by the natural blue light prevalent at this depth in order to prevent polyp retraction, which is promoted by changes in light level in *Goniopora* species.

Representative specimens were then collected and transferred to the IUI laboratory in separate Ziploc bags and stored in black plastic containers for further analyses. Samples of *S*. *pistillata* were collected from a depth range of 1–65 m. We sampled horizontal branches with equal exposure to down-welling light on the upper part and bottom reflectance on the lower part of the branches. The fragments were kept in the IUI flow-through aquarium system inside a dark room until they were photographed and fluorescence spectra were recorded.

### Characterisation of the underwater light field

Downwelling spectral irradiance measurements were conducted using a profiling reflectance radiometer PRR800 (Biospherical Instruments Inc., San Diego, USA) with 19 channel detection (300–900nm) and an integrated Photosynthetically Active Radiation (PAR) sensor. The instrument was deployed at midday (11:00–13:00) on the 29^th^ October 2014 from a boat, using the free fall technique in order to avoid shading or reflectance by the vessel and to keep the light sensor in a vertical position. The instrument was lowered from the boat while its distance from sea bed was kept constant (~1 m) comparing the boat sonar data to the depth sensor data of the instrument. Data were analysed using PROFILER software (Biospherical Instruments Inc., San Diego, USA).

### Depth distribution and fluorescence of prevalent coral species

Fluorescence in shallow water corals (depths between 0.5 and 3 m) was surveyed at night using a blue-light torch and yellow-barrier filter goggles (Nightsea). The reefs in front of the IUI were assessed over a 500 m transect.

Additionally, coral fluorescence was explored during technical SCUBA diving along one belt transect of 50 m^2^ at depths of 2, 5, 10, 15, 40, 50 and 65 m using the underwater fluorescence imaging system for excitation and fluorescence detection. Four localities were explored (Dekel Beach, Oil Jetty, Nature Reserve and IUI) in the Gulf of Eilat/Aqaba during 2010 to 2015. All localities had a fringing reef structure and mesophotic submerged bunks down to a maximal depth of 72 m. Coral identification was achieved by (1) using keys provided by Veron [39,40], Scheer & Pillai [41], Wallace [42] and Hoeksema [43], (2) comparison with the coral collection at the Tel-Aviv University Steinhardt Museum of Natural History and National Research Center, and (3) evaluation by expert coral taxonomists.

### Coral culture

Five to ten fragments from each colony of the studied *E*. *paradivisa* colonies were kept in “light” and “dark” conditions in the flow-through aquarium system at the IUI for one year. Light levels experienced by the “light”-treated fragments were determined with a Li-Cor LI-193SA 4 pi probe attached to a Li-1400 data logger (Li-Cor) at noon on several days over the different seasons. The upper parts of the corals were exposed to maximum fluxes of 10–20 μmol photons m^-2^ s^-1^. The “dark”-treated fragments were kept in complete darkness. At the end of the experiment, the fluorescence of the corals from the different treatments was documented by spectroscopic measurements and photography (see below).

Replicate fragments of a red colour morph of *Echinophyllia* sp. were cultured in the experimental mesocosm of the Coral Reef Laboratory at the University of Southampton [44]. To test the effect of short wavelengths on the coral fluorescence, the five replicate corals were exposed to light with different spectral compositions but with identical photon fluxes of ~20 μmol photons m^-2^ s^-1^ for six months. One group was kept under full spectrum white light (containing violet wavelengths) provided by a metal halide lamp fitted with an Aqualine 10000 burner (13000 K; AquaMedic). The other group was incubated under cyan light (~480nm, lacking violet wavelengths <430 nm) emitted from Aqua Ray Marine Blue LEDs (Tropical Marine Centre).

### Fluorescence photography in the laboratory

Coral fluorescence was photographed through a yellow long-pass filter (Nightsea) upon excitation of the tissue fluorescence with a ~450-nm light source (Nightsea). Standardised conditions (excitation light intensity and exposure times) were established to produce images that allow a comparison of tissue fluorescence intensity.

### Fluorescence spectroscopy

Fluorescence was excited with blue light torches (440–460 nm) and a yellow long-pass filter (Nightsea) to block out the excitation light.

Fluorescence emission spectra were measured with a USB2000 spectrometer and analysed with the SpectraSuite—Spectrometer Operating Software (Ocean Optics Inc.). The background was subtracted and the spectra were smoothed by a running median smoother in SigmaPlot 12 (Systat Software Inc.).

Fluorescence emission spectra of the aquarium-cultured specimens of *Echinophyllia* sp. were recorded with a fibre optic probe coupled to a Cary Eclipse fluorescence spectrometer (Varian). A 0.5 cm spacer attached to the probe was used to measure the fluorescence of comparable areas and enable a quantitative comparison of tissue fluorescence between the experimental samples from the different light treatments.

### Photoconversion under the fluorescence microscope

Following a six-month exposure to cyan light lacking violet wavelengths, the tissue fluorescence of *Echinophyllia* sp. was imaged with an MZ10 Olympus fluorescence microscope equipped with a long-pass GFP filter set (AHF). Green-to-red photoconversion of the fluorescent pigments was induced by local exposure to ~380 nm light for 10 minutes using a UV filter set (AHF). The photoconversion was subsequently imaged using the above mentioned GFP filter set.

## Results

Green fluorescence in shallow water corals was found in most of the colonies surveyed in the reefs in front of the IUI. Notably, essentially no orange-red coral fluorescence was observed with the exception of two orange-red fluorescent individuals, one *Dipsastraea* sp. coral and one corallimorpharian (*Discosoma* sp.), both of which were growing in crevices underneath massive corals.

**Fig 2:**
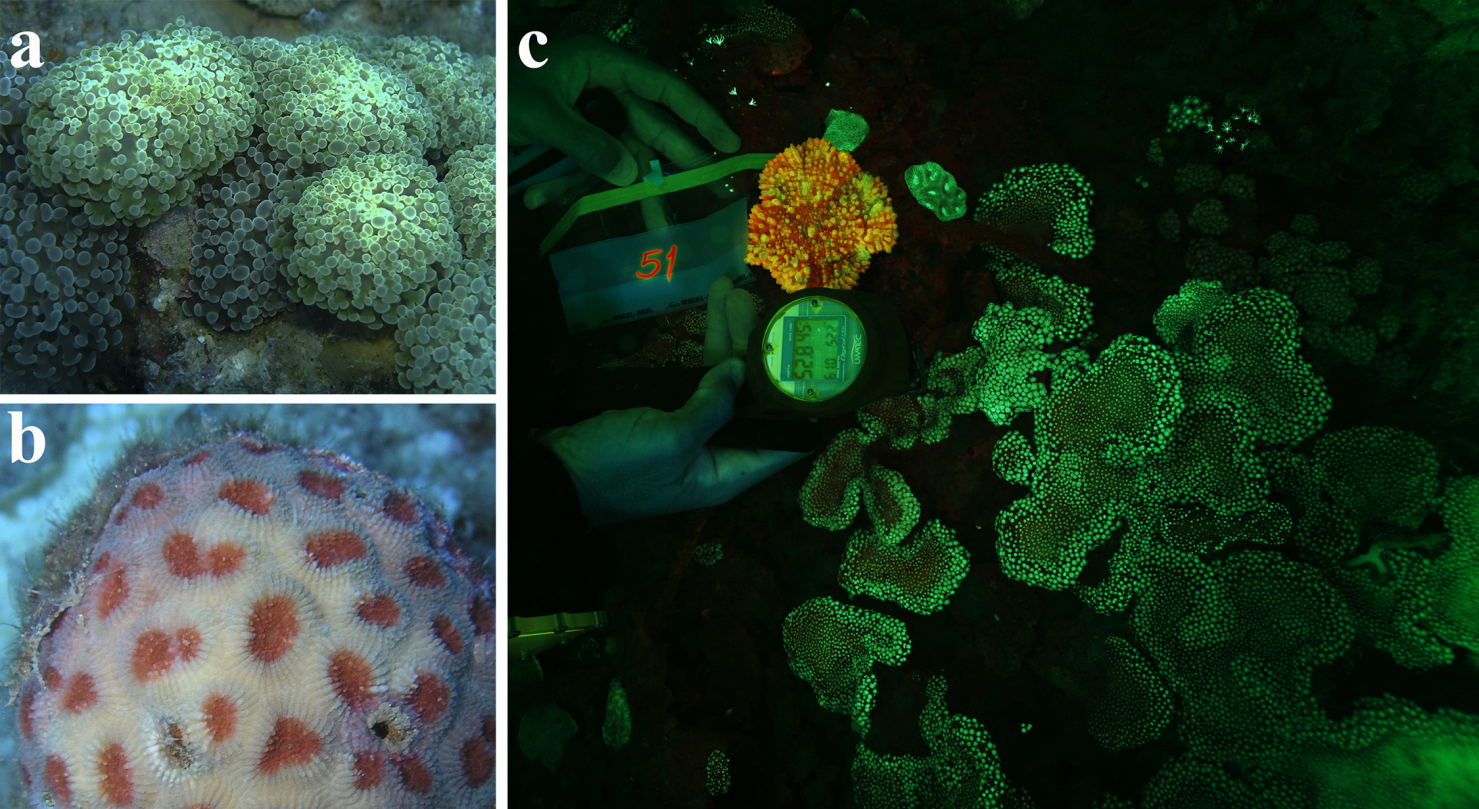
*In-situ* survey of fluorescence in mesophotic corals. (a) Green fluorescence of *Euphyllia paradivisia* and (b) Orange-red fluorescence of *Dipsastraea favus* photographed under excitation by the blue-dominated natural light at depths of ~60 m. (c) Fluorescence imaging system photography showing the orange-red fluorescence emission of *Echinophyllia aspera* and the green fluorescence of *Alveopora ocellata* at ~53 m prior to collection for laboratory analyses.

Visual inspection of corals within the belt transects in depths between 40–65 m revealed that green and orange-red fluorescence excited by the natural, blue-dominated environmental light was widespread and often bright enough to be detected by the naked eye or by a standard underwater camera. The fluorescence imaging system proved helpful in improving the contrast between the fluorescence signal and the background (Fig 2).

### Spectral properties

**Fig 3:**
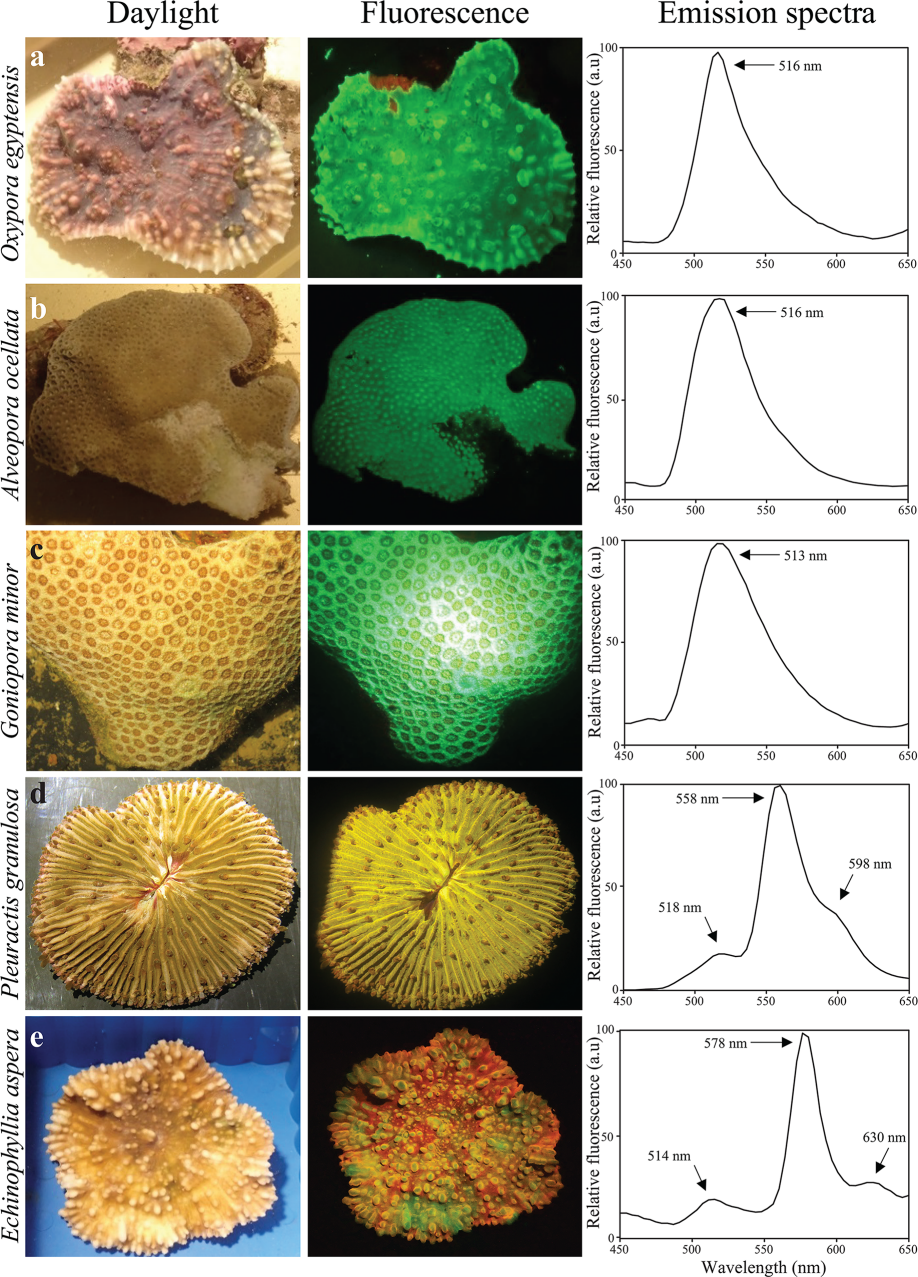
Fluorescence signature of corals collected from mesophotic depths in the Gulf of Eilat/Aqaba. Photographs show the appearance of representative specimens under daylight (left column) or imaged under blue light through a yellow long-pass filter (Nightsea) (middle column). Fluorescence spectra (right column) were recorded after excitation with 450 nm light. Peak positions are indicated by arrows. (a) *Oxypora egyptensis*, (b) *Alveopora ocellata*, (c) *Goniopora minor*, (d) *Pleuractis granulosa* and (e) *Echinophyllia aspera*.

**Fig 4:**
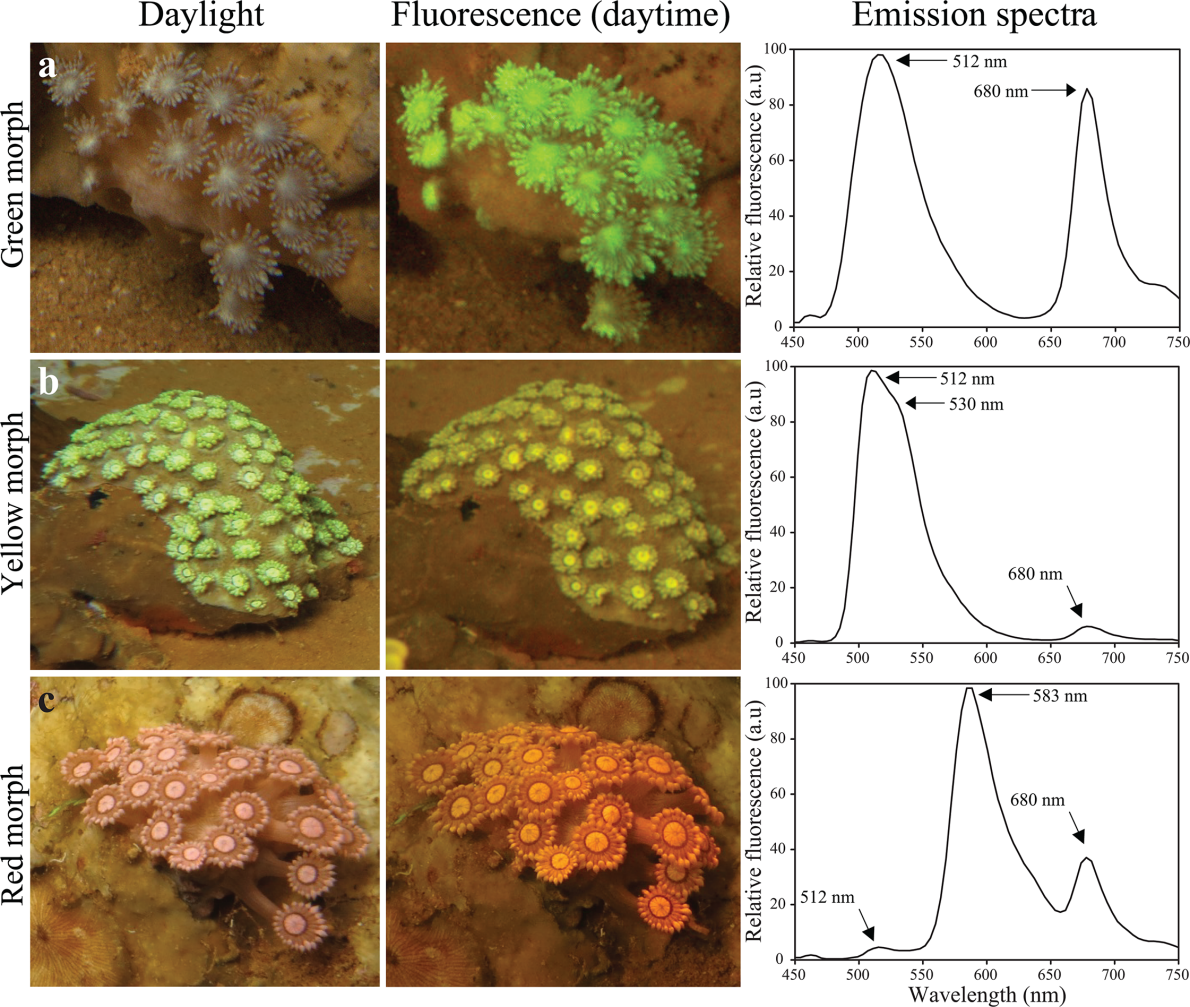
Colour polymorphism of *Goniopora somaliensis*. Representative colonies of the (a) green, (b) yellow and (c) red colour morphs were imaged *in situ* at 50–60 m depth by flashlight photography (left column). Their fluorescence, excited by the natural, down-welling blue-dominated light during daytime was bright enough to be photographed using a yellow long-pass barrier filter (Nightsea) (middle column). Fluorescence emission spectra were recorded under blue light excitation (450 nm) after transfer of the corals to the laboratory (right column). Peak positions are indicated by arrows.

**Fig 5:**
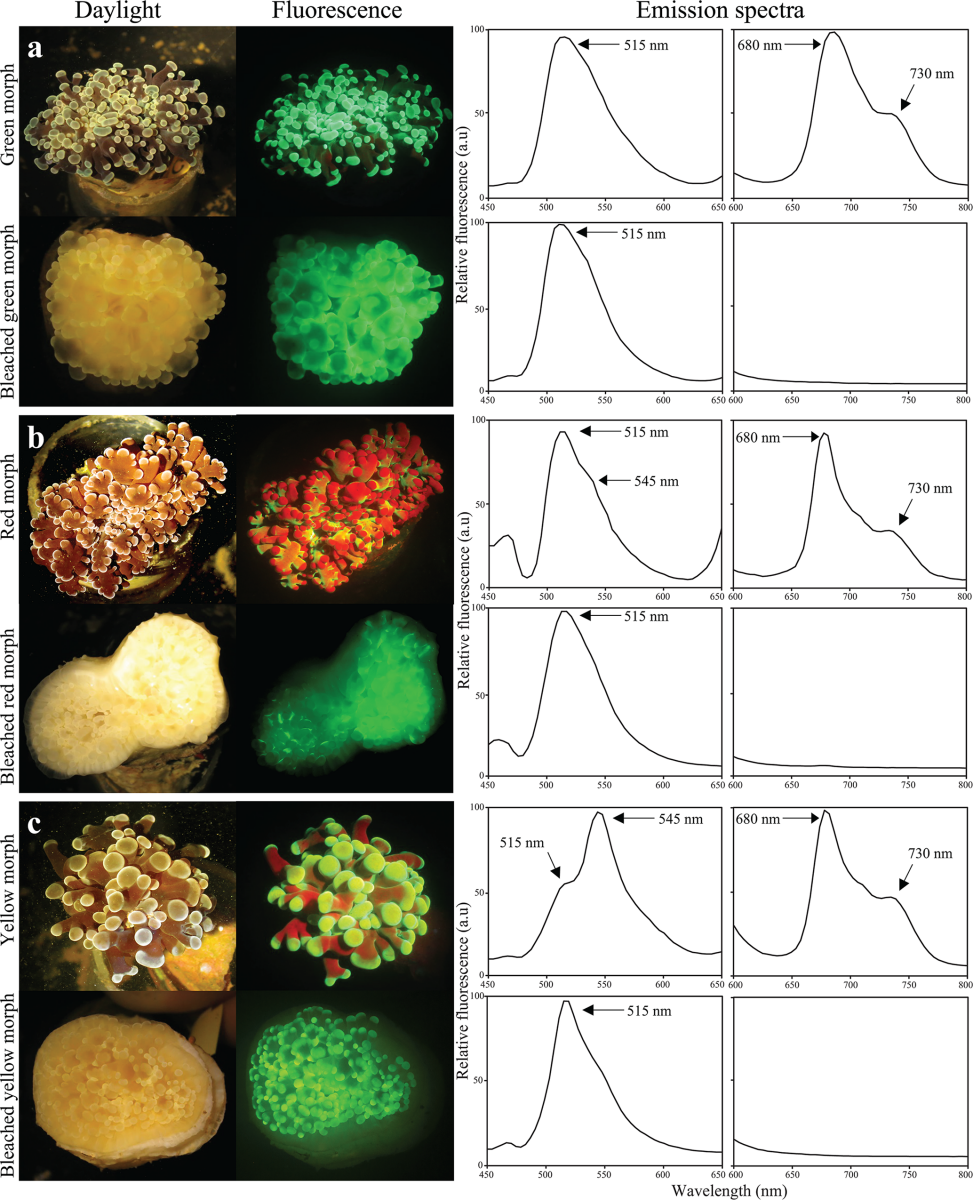
Changes in fluorescence of colour morphs of *Euphyllia paradivisa* from mesophotic depths in response to altered light environments. The daylight appearance and blue-light- induced fluorescence of the green (a), red (b) and yellow (c) colour morphs are imaged after one year of culture under low intensity daylight (upper row) and in complete darkness (lower row). Fluorescence emission spectra in the range of GFP-like proteins (450–650 nm) and chlorophyll (650–800 nm), measured by excitation with ~450 nm, are shown for the displayed individuals. Peak positions are indicated by arrows.

We collected corals from mesophotic depths with representative and visually significant fluorescence in order to gain an overview of the range of the fluorescent pigments present at these depths. After transfer to the laboratory, the fluorescence emission of the living corals was immediately recorded (Figs 3–5, Table 1). Green fluorescence with maxima in the range of 512–522 nm could be found in essentially all examined species. Yellow fluorescence with emission maxima between 522 and 558 nm was detected in more than a third of the species. Emission maxima in the orange-red range from 578 to 584 were recorded in six species. Most of the fluorescence spectra were dominated by a single, narrow peak characteristic of GFP-like proteins [18]. The spectroscopic data of some individuals of *E*. *paradivisa* and *G*. *somaliensis* showed variable contributions of green and yellow fluorescent pigments with overlapping emission spectra.

**Table 1:** Fluorescence emission maxima of corals and corallimorpharians from Eilat reefs.

Species	Collection depth (m)	Green (nm)	Yellow (nm)	Red (nm)	Red sea depth range (m)
**Scleractinian corals:**					
*Acropora* sp.	6	-	-	-	1–60^a^
*Acropora squarrosa*	5,60	**522**	-	-	5–60^a^ ^,^ ^b^
*Alveopora allingi*	50,60	**522**	-	-	20–65^a^
*Alveopora ocellata*	50,60	**516**	-	-	30–65^a^
*Blastomussa merleti*	50	**520**	-	-	5–60^a^ ^,^ ^b^
*Echinophyllia aspera*	53,58	514	535	**578 (**630)	30–60^a^
*Euphyllia paradivisa*	50	**515**	**545**	**-**	36–72^c^
*Favites cf*. *bestae*	60	**512**	-	-	5–60^a^
*Goniopora minor*	50	**513**	-	-	40–65^a^
*Goniopora somaliensis*	55,60	**512**	530	**583**	40–65^a^
*Hydnophora exesa*	6	**512**	-	-	1–25^a^ ^,^ ^b^
*Leptoseris glabra*	60	**516**	-	-	20–72^a^
*Leptoseris* sp.	60	**513**	-	-	20–60^a^
*Oxypora egyptensis*	60	**516**	-	580	30–60^a^
*Pleuractis granulosa*	50	518	**558**	598	40–50^a^
*Pocillopora damicornis*	6	507	**523**	-	1–40^a^ ^,^ ^b^
*Stylocoeniella guentheri*	62	513	524	-	5–65^a^ ^,^ ^b^
*Stylophora pistillata*	1,3,5,10,25,60,65	**516**	-	-	0–65^b^ ^,^ ^d^
*Turbinaria reniformis*	60	**512**	-	-	3–60^a^
**Corallimorpharians:**					
*Rhodactis rhodostoma*	55	**-**	-	584	0.5–65^a^ ^,^ ^e^
*Discosoma unguja*	55	**513**	-	-	0.5–60^a^ ^,^ ^f^

The dominant emission peaks are indicated in bold numbers.

^a^ This study

^b^ WoRMS Editorial Board (2015). World Register of Marine Species. Available from http://www.marinespecies.org at VLIZ

^c^ Eyal et al, unpublished

^d^ [45]

^e^ [46]

^f^ [47]

In *E*. *aspera*, the typical signature of green-to-red photoconvertible proteins was detected [17,32,48]. The emission spectrum shows a local maximum at ~514 nm which can be attributed to the green, unconverted chromophore, and a second peak at ~578 nm with the characteristically pronounced vibronic sideband at ~630 nm, emitted by the red, photoconverted chromophore (Fig 3).

All species showed detectable chlorophyll fluorescence with an emission maximum around ~680 nm. Representative chlorophyll emission spectra in the corals *G*. *somaliensis* and *E*. *paradivisa* are shown in Figs 4 and 5, respectively.

### Fluorescence patterns and colour polymorphisms

Many species collected from depths >50 m were characterised by a uniform distribution of fluorescence over the upper colony surface: e.g. *O*. *egyptensis*, *A*. *ocellata*, *G*. *minor*, *P*. *granulosa* and *E*. *aspera* (Fig 3), while *G*. *somaliensis* was represented by three distinct colour morphs which could be easily distinguished by their green, yellow and orange-red fluorescence (Fig 4). The colour morphs also showed differences in the tissue-specific accumulation of the pigments. Whereas in the green and yellow morphs the fluorescence was concentrated in the polyps, the fluorescent pigments in the red morph were distributed over the whole colony. In *E*. *paradivisa* too, three colour morphs (green, yellow and red) could be distinguished based on the spectral signature and the localisation of tissue fluorescence (Fig 5). In the green and yellow morphs, fluorescence was concentrated in the tips of the tentacles. In the red morph, in contrast, green light emission was evenly distributed over the length of the tentacles and absent from the tentacle tips, which showed instead a dark red fluorescence emitted by the chlorophyll of the zooxanthellae.

**Fig 6:**
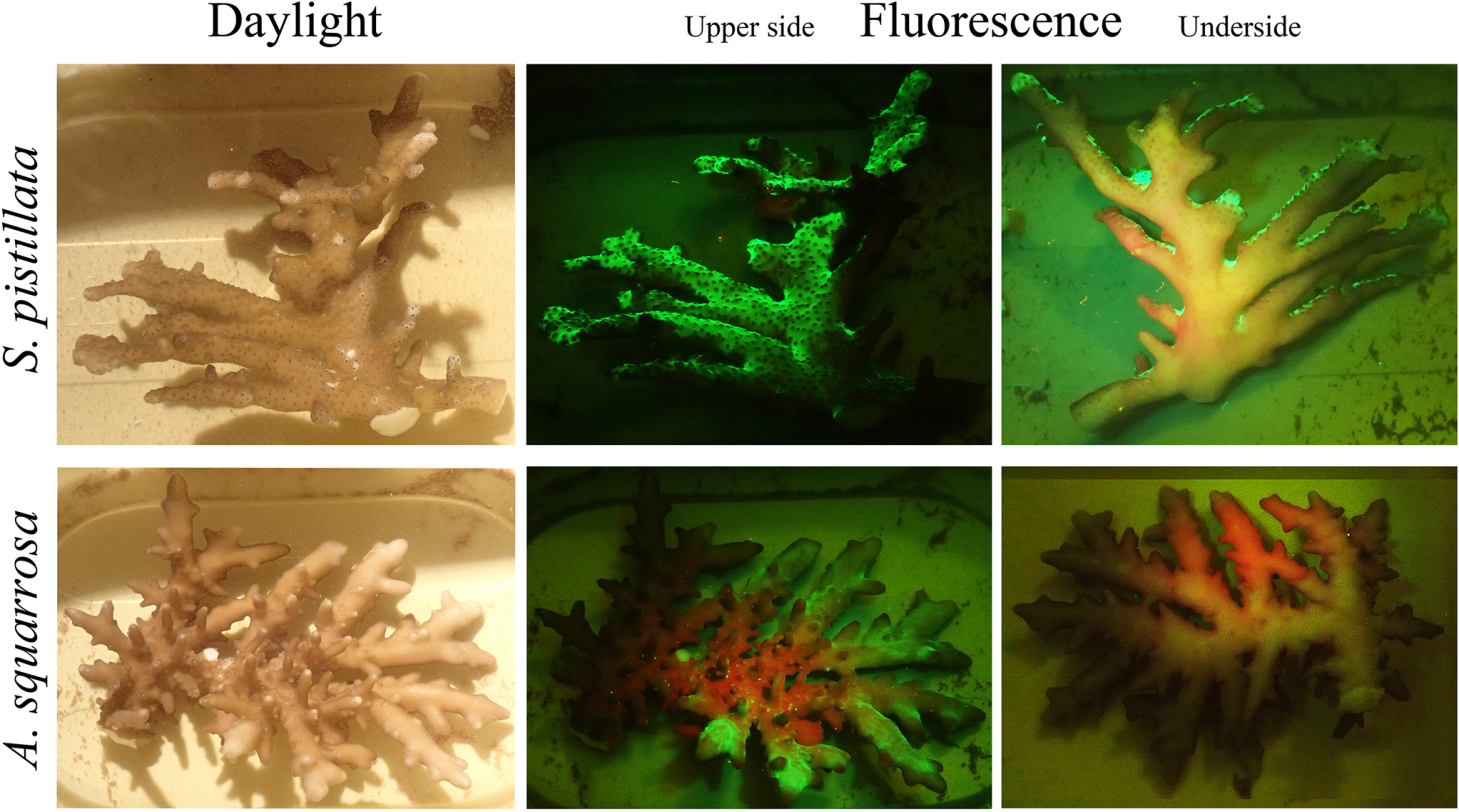
Fluorescence of *S. pistillata* and *A. squarrosa* from depths ≥60 m. Images, taken after the specimens were collected, show the daylight appearance (left column) and the blue-light-induced fluorescence of the upper and underside of the branches (middle/right column).

Deep-water individuals of the branching species *S*. *pistillata* and *A*. *squarrosa* showed a flattened branch morphology. In both cases, the green fluorescence was restricted to the upper part of the branches (Fig 6).

### Light-dependent accumulation of fluorescent pigments

We incubated replicate nubbins of the different colour morphs of *E*. *paradivisa* for 12 months under low-intensity full spectrum daylight and in complete darkness. The photosynthetic pigments of the zooxanthellae were lost completely from the specimens in the dark treatment as evident from the bleached appearance of the corals and lack of the chlorophyll emission peak at ~680 nm (Fig 5). The yellow fluorescence too (emission maximum at ~545 nm) vanished completely. However, under these conditions, the intensity of the green tentacle fluorescence remained essentially unaltered.

**Fig 7:**
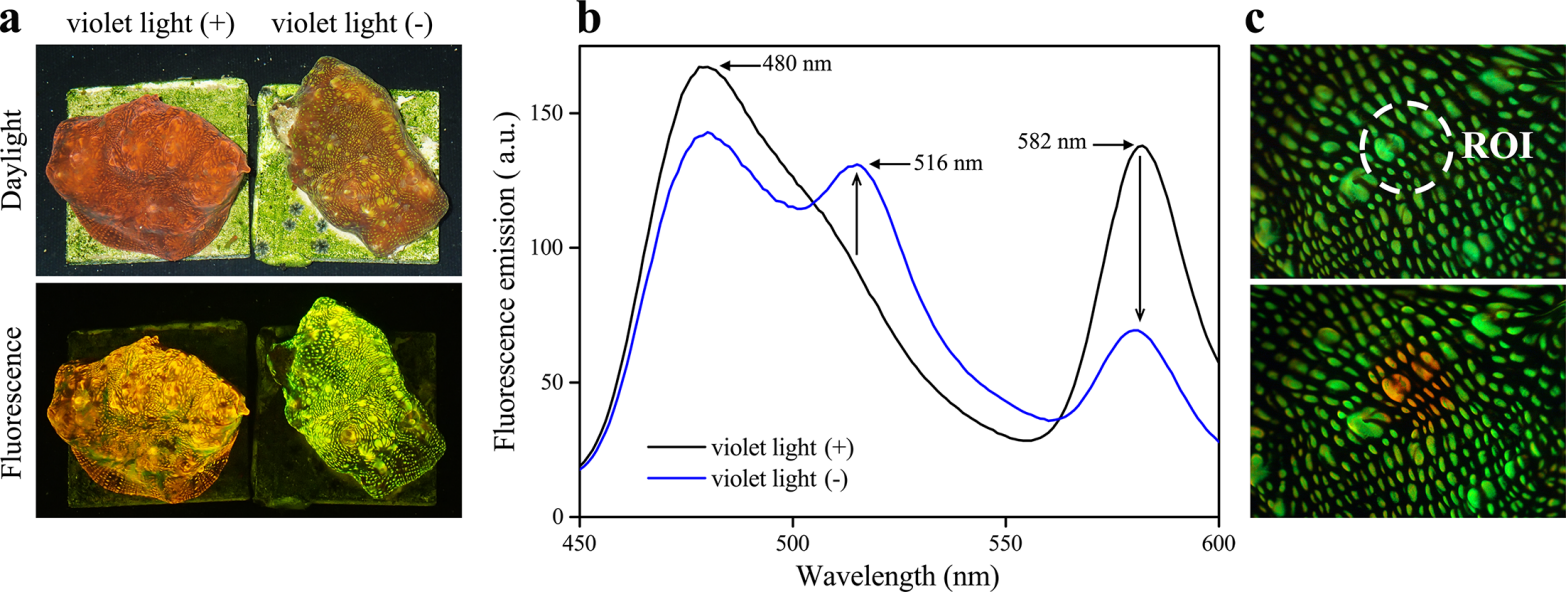
Changes in the tissue fluorescence of *Echinophyllia* sp. in response to experimentally-altered light environments. (a) The daylight appearance (upper panel) and blue-light-induced fluorescence (lower panel) were imaged after 6 months of culture under low intensity, full spectrum white light (violet light [+]) or cyan light (~480 nm, violet light [-]). (b) Fluorescence emission per area upon excitation with 420 nm light was quantified for violet light [+] and violet light [–] treated corals. Spectra represent the means of 10 individual measurements taken from 3 different replicate colonies exposed to the corresponding light environments. Peak positions are indicated by arrows. (c) A violet light [–] treated specimen was photographed with a Leica digital video camera attached to an Olympus fluorescence stereomicroscope using a GFP filter set before (upper image) and after (lower image) exposure of the region of interest (ROI) to focused 380 nm light.

Accumulation of fluorescent pigments was restricted to the upper side of outer branch parts in *S*. *pistillata* and *A*. *squarrosa* collected from ≥60 m depth (Fig 6). Long term experimental exposure of *Echinophyllia* sp. to low intensity of ~480 nm-light, lacking wavelengths <430 nm resulted in a greening of the otherwise orange-red fluorescent corals (Fig 7). Short-term local exposure with light around ~380 nm resulted in a green-to-red photoconversion of tissue fluorescence.

## Discussion

### Spectral properties

Our assessment of coral fluorescence in Eilat reefs in shallow water (depths between 0.5 and 3 m) encountered essentially no yellow (rarely found also in other reef regions [16]) or red fluorescent specimens. In contrast, green, yellow and red fluorescent pigments were found frequently in corals from mesophotic depths ≥50m. Fluorescence at depths >40m is readily visible by eye and can be documented photographically even without optical filter systems owing to the fact that at these depths longer wavelengths are depleted relative to the fluorescence-exciting shorter wavelengths (Fig 1). Thereby, the contrast of the emitted light in the yellow-green and orange-red spectral region is enhanced.

The specific signature of the emission spectrum of *E*. *aspera* with emission maxima at 514 nm and 578 nm identified its dominant pigment as a photoconvertible protein of the class of green-to-red photoconvertible proteins [32,48]. The chromophore of this specific type of GFP-like protein requires exposure to light in the spectral range between 360 to 430 nm (maximal efficiency at 390 nm) in order to undergo the irreversible photochemical reaction that changes the chromophore from a green(514 nm) to a red (578 nm) emitting form [48]. A GFP-like protein with an amino acid sequence with high similarity to the green-to-red photoconvertible EosFP has been isolated from *Echinophyllia echinata* [16].

Several previously studied fluorescent proteins show a reversible increase of their fluorescence intensity and small shifts in the position of their emission maxima with increasing pressure in the range of 0.1–500 MPa [49,50]. The fluorescence intensity of the red fluorescence protein dsRed, for example, was maximally increased by ~8% at a pressure of 200 MPa as compared to normal atmospheric levels [49]. Since the coral pigments in mesophotic depths are exposed to increased pressure of only ~0.5–1.1 MPa, it is unlikely that their spectral properties *in situ* deviate substantially from those determined in our measurements at atmospheric pressure

Our analysis of 16 species of corals (Table 1) from mesophotic depths did not detect a significant contribution of cyan fluorescence with emission maxima in the range of 470–500 nm to the overall fluorescence of these animals. In contrast, cyan fluorescent proteins are frequently encountered in shallow water corals and can dominate the quality of light emission of several species [16,19]. A pronounced cyan fluorescent maximum was detected in the strain of Indo-Pacific *Echinophyllia* sp. that was cultured for the long-term laboratory experiments and in colour morphs of *M*. *cavernosa* [30,32], suggesting that the absence of CFP might not be a uniform feature of corals with a preference for less light-exposed habitats.

### Fluorescence patterns and colour polymorphism

Shallow-water corals display a broad range of fluorescence patterns that result from the tissue-specific expression of GFP-like proteins [51]. Many of the corals from depths >50 m in this work showed a rather homogenous distribution of light emission over the whole colony. Exceptions were found among individuals of *E*. *paradivisa*, which showed a variable localisation of the fluorescent pigments in the tentacles; and among representatives of *G*. *somaliensis*, in which the fluorescence was either distributed evenly over the colony or restricted to the polyps. The latter two species also showed a pronounced colour polymorphism related to variations in the expression of the dominant fluorescent pigment, a phenomenon that is common among anthozoans [30,32,52–54]. As demonstrated for the coral *Acropora millepora*, these colour polymorphisms can be the result of the pigment-encoding genes being present in multiple copies [55]. Depending on how many genes are active, the corals will become more or less colourful. In shallow waters, where the GFP-like proteins can fulfil a photoprotective function, this genetic framework can enable corals to invest either in expensive high-level pigmentation, offering benefits under light stress, or to rely on low tissue pigment concentrations and use the conserved resources for other purposes, a preferable strategy in low light-exposed environments.

### Regulation of pigment production

Previous studies showed that the expression of GFP-like proteins in shallow water corals is often upregulated at the transcriptional level by the light intensity, specifically by blue light [19]. At low-light intensities, the tissue fluorescence decreases, usually to almost undetectable levels [19,24].

In contrast, corals such as *L*. *hemprichii* or *M*. *cavernosa*, which frequently occupy less light-exposed habitats showed a constitutive expression of their fluorescent pigments [31,32].

We incubated *Echinophyllia* sp. and *E*. *paradivisa* under reduced light intensity to examine whether the constitutive expression of fluorescent pigments was a common phenomenon in corals from low-light environments.

Long-term experimental exposure of *Echinophyllia* sp. to low intensity of cyan light (~480 nm), which lacked wavelengths <430 nm resulted in a colour change from orange-red to green. This greening could be reversed by a brief, local exposure to short wavelength light (~380 nm). A comparable response was previously observed for the red fluorescent protein mcavRFP in the tissue of *M*. *cavernosa*, suggesting that the exposure to short-wavelength light is essential for these corals to develop their dominant red tissue fluorescence [31]. Since the production of the pigment in its native green state is sustained at low light levels [31], the relevant genes are most likely constitutively expressed and light is only required for the post-translational modification of the pigments. The *E*. *aspera* specimens analysed in the present study showed bright orange-red fluorescence *in situ* at a depth of >50 m, indicating that even at this depth, the underwater light field contains sufficient amounts of photons in the short—wavelength range to induce efficient photoconversion.

Variable responses were observed after prolonged incubation of the different colour morphs of *E*. *paradivisa* in darkness. All the morphs completely lost the chlorophyll fluorescence of the zooxanthellae. Accordingly, the prevailing red emission of the red morph was lost. In contrast, in all the morphs, the green fluorescence with an emission maximum at ~515 nm persisted essentially unaltered in corals cultured in the dark. This indicates that the relevant genes are constitutively expressed.

Interestingly, the 545 nm-peak of the yellow fluorescent pigment (most pronounced in the yellow morph) was not detected in the spectra measured after dark incubation. This may indicate that this pigment is either a yellow fluorescent protein with a light-dependent expression or that it represents a derivative of the green fluorescent emitter that is formed in a light-dependent reaction. Further studies will elucidate the putative mechanism responsible for this observation.

The restricted accumulation of green fluorescent pigments in the upper-side of *S*. *pistillata* and *A*. *squarrosa* branches collected from ≥60 m depth suggests an influence of the irradiating light on the regulation of pigment production. This localised accumulation in the most light-exposed branch parts has been frequently observed for light-regulated GFP-like proteins in shallow water corals, in particular among acroporids [24,29].

### Biological function of fluorescence at mesophotic depths

Recent studies have demonstrated the ability of both FPs and CPs to fulfil a photoprotective function for the zooxanthellae in some shallow-water corals by screening photosynthetically-active radiation (PAR) [24,29]. In line with their function, these photoprotective pigments are tightly controlled by the light environment. The need for photoprotection can be ruled out for corals from mesophotic reefs and may explain the absence of dominant cyan fluorescence at greater depths, since the absorption properties of these pigments appear to be well suited for screening of algal chlorophyll [19]. Nevertheless, species such as *S*. *pistillata* with a broad depth distribution ranging from very shallow water to mesophotic depths (Table 1), may still reveal the light-mediated regulation mechanisms associated with the photoprotective function that fluorescence fulfils in the shallow water population.

We have found that a number of brightly fluorescent species, including *E*. *aspera* and *E*. *paradivisa*, have their maximal abundance at depths deeper than 30 m. The production of individual pigment molecules might be considered comparably cheap [31], since only a single gene is required to produce the functional FP and their turnover is slow, presumably due to the stable beta-can fold. However, the high tissue concentrations of up to 7–14% of the total soluble protein [31,32] can only be reached by the simultaneous expression of multiple copies of the same gene [24]. Hence, maintaining high tissue fluorescence is energetically costly, and it is therefore very likely that the fluorescent pigments in mesophotic corals have a particular biological function that differs from photoprotection. This assumption is supported by the light-independent, constitutive expression of their FP genes, which is atypical for the photoprotective pigments from shallow water corals [31,32]. Furthermore, in contrast to shallow water corals, yellow and orange-red fluorescence were more abundant in the species with a preference for greater depths (Table 1). Also *Leptoseris* spp. from Hawaii showed a shift of the dominant FPs from shorter (cyan) in shallow waters to longer (green) wavelengths in mesophotic depths [15]. The trend that fluorescence in mesophotic corals tends to be red-shifted compared to the shallow water representatives further points to a distinct biological function in corals from deeper habitats. Alternative functions of FPs, which have been discussed, include modulation of the activity of regulatory photosensors analogous to phytochromes and cryptochromes of higher plants [56], links to visual ecology of the reef fishes [57,58] and PAR enhancement [12]. Future experimental studies are required to confirm the function of fluorescence in mesophotic corals.

### Coral reef monitoring

Coral fluorescence can be detected with a high signal-to-background ratio and has been suggested as an indicator of coral health and live coral cover in reef-monitoring approaches [19,20,59–61]. Indeed, the ability to detect coral recruits *in situ* is increased by screening for their fluorescence [62,63]. The use of advanced wide field-of-view fluorescence imaging shows the potential of coral fluorescence to be used in semi-automated image analysis for habitat mapping [37]. Since coral fluorescence in mesophotic reefs appears to be widespread, bright and spectrally diverse, fluorescence imaging represents a promising approach for the monitoring of mesophotic reefs.

### Bio-prospecting

Fluorescent proteins of the GFP-family have revolutionised biomedical research, as *in vivo* labels of proteins or markers of gene expression [64,65]. The range of differently coloured FPs isolated from marine invertebrates has facilitated multicolour imaging of biological processes in live cells, while photoconvertible proteins have enabled advanced tracking of dynamic processes in living cells and organisms, such as protein movement [66,67]. Furthermore, they are also essential tools for super-resolution microscopy [68]. Red fluorescent proteins are particularly desirable for imaging application, since their detection is facilitated by the better penetration of cells and tissues by long-wavelength light and reduced cellular autofluorescence in the red emission range [69]. The high number of long-wave emitting FPs that we have encountered in the present study suggests that mesophotic reefs may hold novel marker proteins useful for biomedical research applications.
